# Cytotype distribution and chloroplast phylogeography of the *Actinidia chinensis* complex

**DOI:** 10.1186/s12870-021-03099-y

**Published:** 2021-07-06

**Authors:** Zhi Wang, Caihong Zhong, Dawei Li, Chunlin Yan, Xiaohong Yao, Zuozhou Li

**Affiliations:** 1 CAS Key Laboratory of Plant Germplasm Enhancement and Specialty Agriculture, Wuhan Botanical Garden, The Chinese Academy of Sciences, Wuhan, 430074 Hubei China; 2 College of Life Sciences, University of Chinese Academy of Sciences, Beijing, 100049 China

**Keywords:** Phylogeography, Polyploidy, Genetic diversity, Ecological niche models, Subtropical China, *Actinidia chinensis* complex

## Abstract

**Background:**

Plant phylogeographic studies of species in subtropical China have mainly focused on rare and endangered species, whereas few studies have been conducted on taxa with relatively wide distribution, especially polyploid species. We investigated the cytotype and haplotype distribution pattern of the *Actinidia chinensis* complex, a widespread geographically woody liana with variable ploidy in subtropical China comprising two varieties, with three chloroplast fragments DNA (*ndh*F-*rpl*132, *rps*16-*trn*Q and *trn*E-*trn*T). Macroevolutionary, microevolutionary and niche modeling tools were also combined to disentangle the origin and the demographic history of the species or cytotypes.

**Results:**

The ploidy levels of 3338 individuals from 128 populations sampled throughout the species distribution range were estimated with flow cytometry. The widespread cytotypes were diploids followed by tetraploids and hexaploids, whereas triploids and octoploids occurred in a few populations. Thirty-one chloroplast haplotypes were detected. The genetic diversity and genetic structure were found to be high between varieties (or ploidy races) *chinensis* and *deliciosa*. Our results revealed that these two varieties inhabit significantly different climatic niche spaces. Ecological niche models (ENMs) indicate that all varieties’ ranges contracted during the Last Inter Glacial (LIG), and expanded eastward or northward during the Last Glacial Maximum (LGM).

**Conclusions:**

Pliocene and Plio-Pleistocene climatic fluctuations and vicariance appear to have played key roles in shaping current population structure and historical demography in the *A. chinensis* complex. The polyploidization process also appears to have played an important role in the historical demography of the complex through improving their adaptability to environmental changes.

**Supplementary Information:**

The online version contains supplementary material available at 10.1186/s12870-021-03099-y.

## Background

In the last several decades, many phylogeographic studies have focused on the Qinghai-Tibetan Plateau and adjacent areas (the Himalayas and Hengduan Mountains) in China [1–4]. As an area harboring many ancient endemic genera and families, the subtropical region in China has recently attracted much attention due to its complex topography and fluctuated paleoclimate [5–7]. Nevertheless, most of these phylogeographic studies in these two hotspots of China have mainly focused on rare and endangered species [8–11], with few cases examined in the species with a relatively widespread range [12, 13], especially for polyploid species. Polyploidization is a common phenomenon in higher plants and plays an important role in plant diversification and adaptation to novel environments [14]. Although the wide recognition of the prevalence of polyploidy and accumulative cytological evidence for recent whole-genome duplication(s) in many plant groups [15, 16], studies on the phylogeography of polyploid species in these two hotspots in China [17–19] are few relative to those in other hotspots in the world (e.g. North America and Alps in Central Europe).

The *Actinidia chinensis* complex is a geographically widespread woody liana with variable ploidy in subtropical China. It is widely planted around the world as an important horticultural fruit tree crop. In the newly revised infrageneric system of *Actinidia,* the complex comprises three varieties, *A. chinensis* var. *chinensis* (hereafter: var*. chinensis*), *A. chinensis* var. *deliciosa* (hereafter: var. *deliciosa*), and *A. chinensis* var. *setosa* (hereafter: var*. setosa*) [20]. However, phylogenetic studies in *Actinidia* based on chloroplast DNA sequences indicate that var. *setosa* should not be included in *A. chinensis* complex but rather treated as a separate species (*A. setosa*) [21]. Previous studies have shown that the vast majority of var*. chinensis* and var. *deliciosa* can be distinguished by morphological characteristics such as the presence of overwintering buds or the hair types covering on flowering branchlets and fruits [22]. However, there are still morphological intermediates of these two varieties identified in their area of overlap in central China [23–25]. *Actinidia chinensis* var*. chinensis* grows mainly in eastern and central China, whereas var. *deliciosa* is more inland in central and western China [26]. They coexist in many localities of the overlap region but are slightly separated vertically with var. *chinensis* being found at lower elevations. A cytogeographic analysis has revealed that ploidy types of the *A. chinensis* complex vary from diploid to hexaploid and that vars. *chinensis* and *deliciosa* comprise different cytotypes (var*. chinensis*: 2 × and 4x, var. *deliciosa*: 6 × with few 4x) [25]. The existing population genetic studies of the *A. chinensis* complex have been based on few sample sets and do not cover the entire geographic range of this species [22, 24, 25, 27]. Thus, the genetic diversity and population divergence history of the *A. chinensis* complex and correlations based on ploidy level are still unclear.

The development of ecological niche models (ENMs) is a more popular tool to predict the changes in species distribution in response to past or future climatic events and which has been used widely in recent phylogeographic studies [28]. In combination with the methods of molecular phylogeography, ENMs can help to reveal the processes that have shaped current distribution patterns of populations and species [29, 30].

In this study, flow cytometric measurement (FCM) [31] was used to identify cytotypes of the *A. chinensis* complex, and cpDNA markers were used to investigate the genetic diversity and population structure of the *A. chinensis* complex. We used mismatch distribution analysis (MDA) together with ENMs to investigate the evolutionary history and demographic structure. The main objectives of our study were to (a) reveal the pattern of cytotype distribution; (b) evaluate the level of genetic diversity and the degree of genetic differentiation among populations for each variety; and (c) test the origin of polyploidy and explore the demographic history of the *A. chinensis* complex. Our aim was to explore patterns and mechanisms of population diversity and historical demography of a typical polyploidy plant in the subtropical forest that generally correspond to the Metasequoia Flora (Sino-Japanese Kingdom), one of the two major floristic regions in the East Asian Floras [7].

## Results

### Cytotype diversity and distribution

Ploidy was estimated for 3,338 individuals with fresh leaves harvested from hydroponic shoots as 248 individuals failed to obtain fresh leaves. Five ploidy types (di-, tri-, tetra-, hexa- and octoploid, Additional file 1: Figure S1) were identified and the frequency of these ploidy types were 51.56%, 0.48%, 22.65%, 25.22% and 0.09%, respectively (Additional file 2: Table S1). All the diploids belonged to var*. chinensis*, whereas all the hexaploids belonged to var. *deliciosa*. The tetraploids were shared between both var. *chinensis* and var. *deliciosa*. Sixteen triploids having similar morphological characteristics with var. *chinensis* were observed in the diploid, tetraploid, and mixed populations (2x, 4x), whereas rare octoploids were found in two hexaploid var. *deliciosa* populations and one mixed population (2x, 4x, and 6x), respectively.

**Fig. 1:**
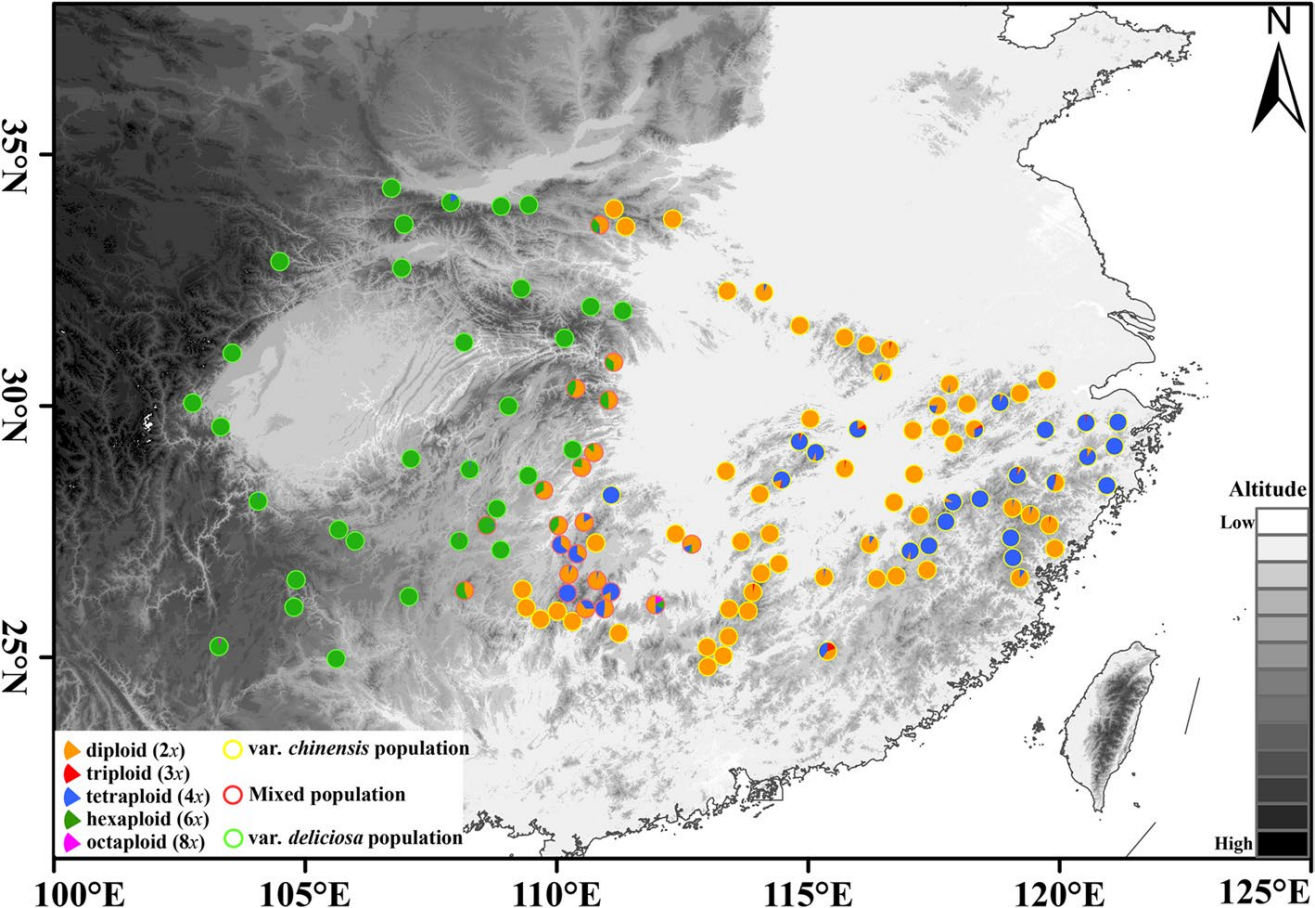
Cytotype distribution of the *A. chinensis* complex. Each location is represented by a filled circle or pie chart, with ploidy levels indicated by color. Map generated in ESRI ArcGIS 10.3

Diploid var*. chinensis* and hexaploid var*. deliciosa* exhibited clear spatial segregation from east China to west China, and the main distribution of the two varieties overlaps in mountainous regions of midwestern China (Fig. 1). The tetraploid distribution area was mainly in the Wuyi and Mufu mountains in east China and Xuefeng mountain in the midwestern China. The subsequent phylogenetic analysis also showed that two parts of the tetraploids belonged to two different lineages (Fig. 3a) which have differentiated at 2.22 Ma.

### Genetic diversity and population structure

**Fig. 2:**
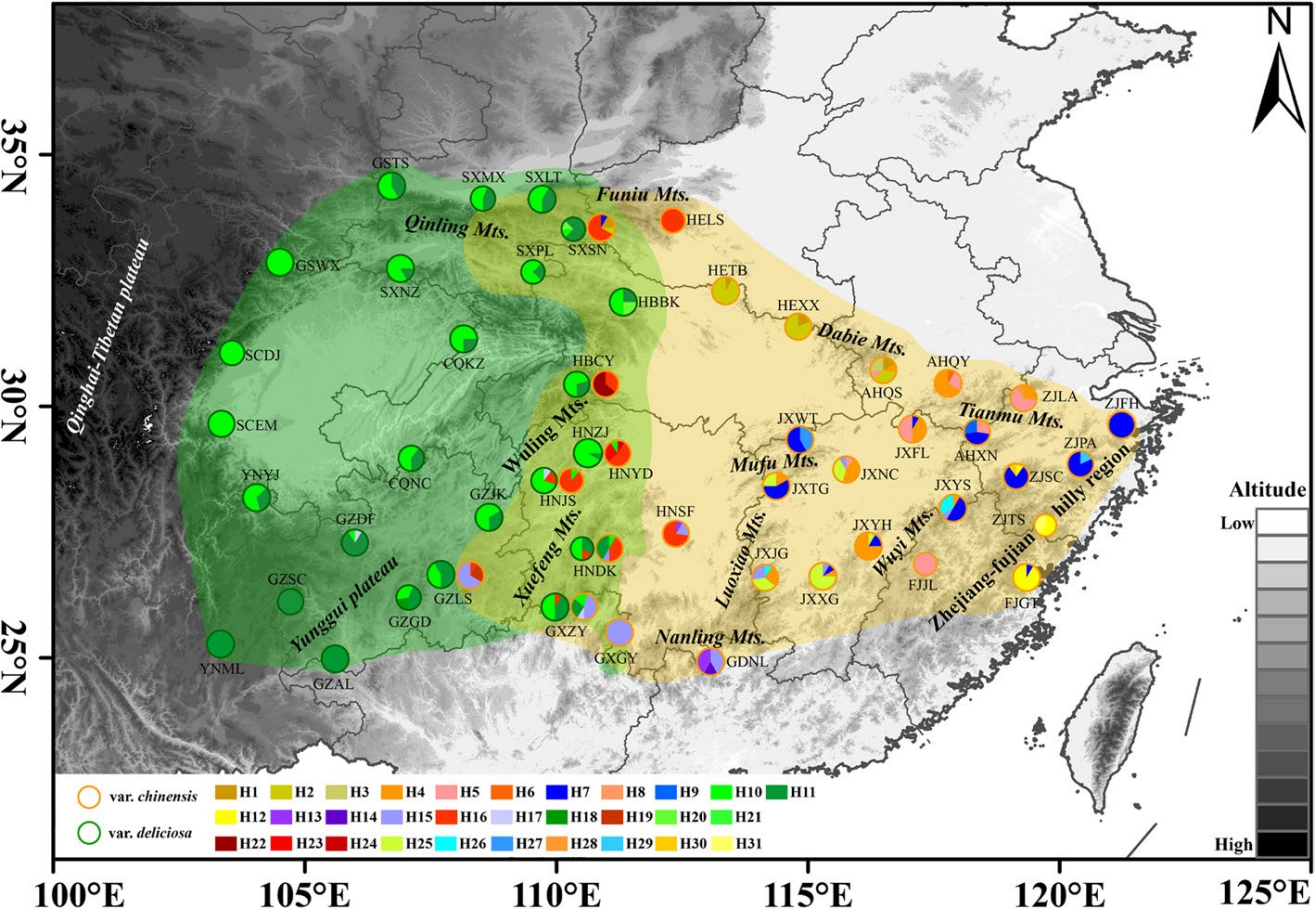
Geographical distribution of cpDNA haplotypes. For the *A. chinensis* complex, geographical locations of the 50 populations and distributions of 31 chloroplast haplotypes of the *A. chinensis* complex examined in this study. The populations of var. *chinensis* and var. *deliciosa* are indicated different color. Map generated in ESRI ArcGIS 10.3

To investigate the genetic diversity and population structure, three cpDNA fragments (*ndh*F-*rpl*132, *rps*16-*trn*Q, and *trn*E-*trn*T) across 608 sampled individuals (Additional file 2: Table S2) from 50 populations were successfully sequenced. The total length of the concatenated sequence was 1,854 bp. A total of 35 polymorphic sites and 31 haplotypes were identified (Additional file 2: Table S3). Twenty-eight haplotypes were present in var*. chinensis* and three haplotypes in var*. deliciosa*. Four haplotypes were shared vars*. chinensis* and *deliciosa* (Fig. 2, Additional file 2: Table S4). The total genetic diversity of var*. chinensis* (*H*_T_ = 0.920, *H*_S_ = 0.470) was higher than that of var. *deliciosa* (*H*_T_ = 0.552, *H*_S_ = 0.380). The coefficient of genetic differentiation *N*_ST_ was significantly larger than *G*_ST_ for var*. chinensis*, and vice versa for var*. deliciosa* (Table 1).

**Table 1:** Genetic diversity and differentiation analyses for cpDNA variations in var. *chinensis* and var. *deliciosa*

Species	*H*_S_	*H*_T_	*G*_ST_	*N*_ST_
*A. chinensis*	0.470	0.920	0.490	0.591**
*A. deliciosa*	0.380	0.552	0.312	0.252
Pooled	0.441	0.884	0.501	0.638**

^**^ Indicates that *N*_ST_ is significantly different from *G*_ST_ (*P* < 0.01)

No spatial population structure was detected by the SAMOVA analysis of cpDNA haplotypes from 50 populations; the *F*_CT_ values did not have significant peaks as the value of *K* increased from 2–20 (Additional file 1: Figure S2). Therefore, the populations were divided into two groups by taxonomy for the AMOVA analysis. The AMOVA analysis (Additional file 2: Table S5) revealed that 25.06% of the molecular variation was distributed between varieties (*F*_CT_ = 0.251, *P* < 0.01). The inter-variant population fixation indices (*F*_ST_) were 0.654 and 0.352 (*P* < 0.01) for var*. chinensis* and var*. deliciosa*, respectively. The isolation-by-distance (IBD) tests based on the pairwise *F*_ST_/(1- *F*_ST_) genetic distances against the geographic distances detected no significant correlation for the cpDNA (*R*^2^ = 0.1234, *P* = 0.988) among the 50 populations of the *A. chinensis* complex.

### Phylogenetic relationships and divergence dating

**Fig. 3:**
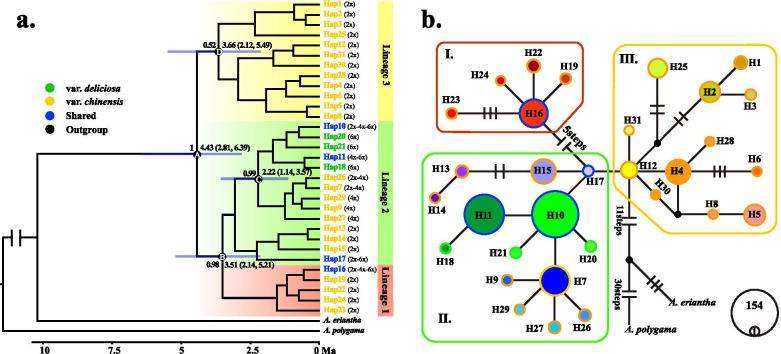
BEAST-derived chronograms and haplotypes network of the *A. chinensis* complex. **a** BEAST-derived chronograms of the *A. chinensis* complex based on cp DNA sequences. Blue bars indicate 95% HPD clade credibility intervals for nodes of particular interest with ages (in Ma). The cytotypes were also labeled for each haplotype. **b** The network of 31 chloroplast haplotypes. Each circle corresponds to a unique haplotype, with circle size reflecting its frequency. Small black circles indicate missing haplotypes

Phylogenetic analysis of the concatenated cpDNA haplotype data sets recovered three haplotype lineages (lineages 1, 2, and 3; Fig. 3a), a result consistent with the phylogenetic network based on the 31 haplotypes (Fig. 3b). Phylogenetic relationships of the haplotype lineages broadly correspond to distinct geographical regions (Figs. 2 and 3). The haplotypes in lineage 1 were mainly found in mountainous regions of midwestern China where the two varieties overlap. In lineage 1, the haplotype H16 was shared by var*. chinensis* and var*. deliciosa*, but other haplotypes only belong to the diploid population of var*. chinensis*. The haplotypes in lineage 3, which belong to var*. chinensis* (2x) were found in eastern China, such as the Wuyi mountains, Dabie mountains, and Luoxiao mountains. Finally, almost all polyploid haplotypes were found in lineage 2. The dominant haplotype H10 and H11 occurred in 154 and 111 individuals, respectively. Although these two haplotypes were shared by var*. chinensis* and var*. deliciosa*, they still mainly belong to var*. deliciosa*. Nearly half of the haplotypes (16/31) were restricted to a single population. For example, in lineage 3, H3, H6, and H8 were found exclusively in Dabie mountain and Huang mountain, whereas H28, H30, and H31 appeared only in the Zhejiang-Fujian Hilly Region.

The time-calibrated evolutionary tree (Fig. 3a) showed that the divergence time between the *A. chinensis* complex and the outgroup (*A. eriantha*) dates back to an estimated 11 million years ago (Ma) during the late Miocene. For the 31 cpDNA haplotypes of the *A. chinensis* complex, the mean crown-group age of the *A. chinensis* complex was ca. 4.43 Ma (95% HPD 2.81–6.39 Ma, node A), thereby indicating the recent diversification of the cpDNA haplotypes of the *A. chinensis* complex during the Pliocene. Two polyploid haplotype lineages diverged at ca. 2.22 Ma (95% HPD 1.14–3.57 Ma, node C).

### Demographic history and ancestral geographical area

All populations were divided into four groups E2x, E4x, M2x, and M4 × 6x which represent the eastern diploid populations, eastern tetraploid populations, midwestern diploid populations, and midwestern tetraploid and hexaploid populations, respectively. The mismatch distributions of group E4x and M4 × 6x were unimodal (Additional file 1: Figure S3), and the *F*s test and Tajima’s *D* test of group E4x and M4 × 6x were also negative, although only the tests for group E4x were significant (*P* < 0.05, Table 2), closely fitted to the expected distribution under the sudden expansion model. For the other two groups (group M2x and E2x), there were no explicit signals of population expansion or equilibrium, as evidenced from the neutral tests and mismatch analysis (Table 2; Additional file 1: Figure S3), although the variance of the sum of square deviations and Harpending’s raggedness index tests (*SSD* and *HRI*, *P* > 0.05; Table 2) failed to reject the spatial expansion model. The tau values (τ) presented a rough estimate when population expansion began. The approximate expansion times (year) for groups E4x and M4 × 6x were measured as 0.084 (0.033–0.136) Ma BP, 0.137 (0.081–0.162) Ma BP, respectively.

**Table 2:** Population demography of different groups for the *A. chinensis* complex

Group	Parameter(τ)	(*t,*Ma)	*SSD*	*HRI*	Tajima’s *D*	Fu’s *F*_*S*_
Group E2x	5.830 (2.477–10.316)	NC	0.060	0.194	0.920	2.731
Group E4X	0.446 (0.177–0.724)	0.084 (0.033–0.136)	0.002*	0.178	-1.663*	-3.104*
Group M4 × 6x	0.729 (0.432–0.861)	0.137 (0.081–0.162)	0.031*	0.215	-1.230*	-1.408
Group M2x	3.928 (2.027–6.203)	NC	0.046	0.134	0.484	0.926

*SSD* Sum of squared deviation, *HRI* Harpending’s raggedness index, Tajima’s *D* and Fu’s *F*_S_ are neutral test indices, *NC* Not calculated

^*^P < 0.05

**Fig. 4:**
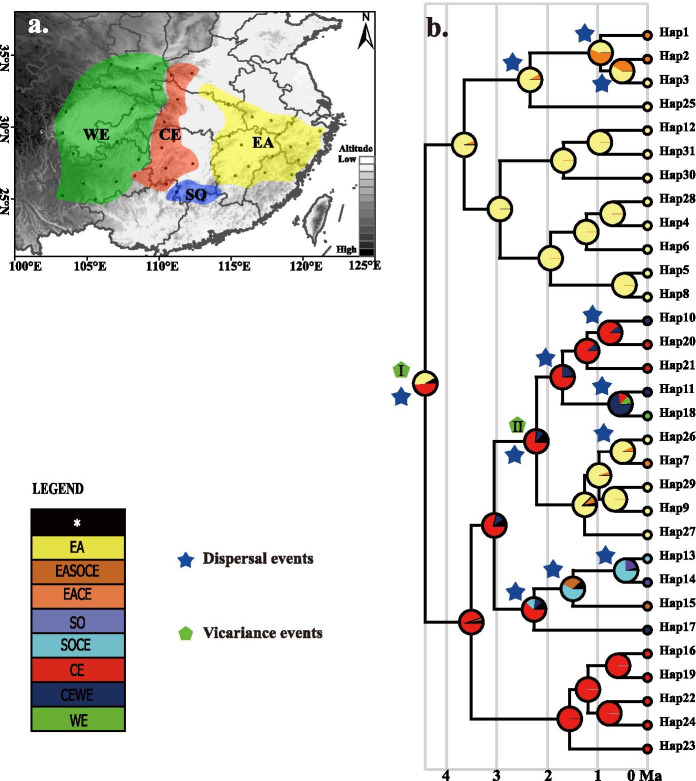
Ancestral areas, reconstructed with the Bayesian Binary MCMC method

The BBM (Bayesian Binary MCMC) analysis of ancestral distribution areas (Fig. 4) revealed two vicariance events and twelve dispersal events. As based on the topology of the intraspecific chronogram, a likely ancient vicariance event (node I, EA, and CE) of two sister lineages was recovered. A subsequent vicariance event (node II) between populations of tetraploid var. *chinensis* in eastern China (EA) and tetraploid/hexaploid var. *deliciosa* in central-western (CE and WE) China was also recovered. Recent colonization events from the eastern region (EA) to the central region (CE) were inferred, as based on the genetic admixture (H1, H2, and H7) in the adjacent mountains (e.g. Qinling and Dabie Mountains) or between the distant mountains (e.g. Qinling and Wuyi Mountains), with multiple expansions at different times (Fig. 4). For the diploid cytotypes, the mountains of eastern China (EA) were inferred as the most likely ancestral area. The ancient haplotypes (H12, Fig. 3b) identified in haplotype network analysis were also distributed in populations ZJTS and FJGT of eastern China. However, for the polyploid varieties, the mountains of central China (CE), e.g., Xuefeng mountain were deduced as the most likely ancestral area, which was also supported by the results of ENM analysis. No extinction events were discovered in any lineage by BBM analysis.

### Ecological niche modeling and niche comparison

**Fig. 5:**
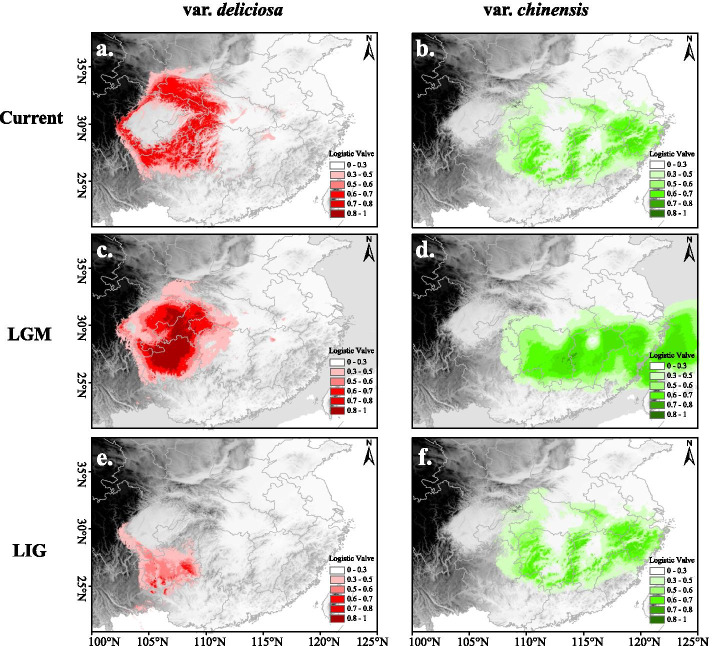
Potential distributions of var. *chinensis* and var. *deliciosa* predicted with MaxEnt. shown at 2.5 arc minute resolution and projected from six bioclimatic variables representing the current (**a**, **b**), LGM (**c**, **d**), and LIG (**e**, **f**) climatic conditions, respectively. Warmer colors denote areas with a higher probability of presence

The values of Area Under the Curve (AUC) between 0.7 and 0.9 indicate good discrimination. The AUC values of ENM for each variety and climate scenario were high (0.978/0.982), indicating that all models performed well in predicting the suitable habitat for each variety. The current distributional predictions were good representations of the actual distribution of both varieties. The main geographic distributional areas for both varieties were predicted mainly in the mountains of the subtropical region in China. The elevations predicted for var*. deliciosa* was often higher than that of var. *chinensis*. The geographic areas of the two varieties overlapped in the mountainous regions of midwestern China. The predicted geographic range for var*. deliciosa* during the LIG (LIG, Last Inter Glacial period) was more southward than that of the present (Fig. 5a, e). The variety var. *chinensis* also had a more restricted distribution during the LIG than that in the present, and the range was more broken than currently (Fig. 5b, f). The predicted species distribution during the LGM for vars. *chinensis* and *deliciosa* were both significantly larger than the predicted distributions under the LIG or current conditions (Fig. 5), suggesting that the distribution ranges of the two varieties had expanded during the LGM.

**Fig. 6:**
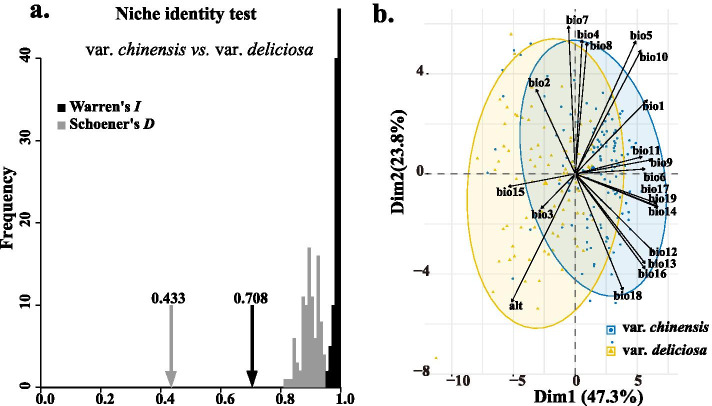
Niche comparison of var. *chinensis* and var. *deliciosa*. **a** Niche-identity tests by comparing the niches of var. *chinensis* and var*. deliciosa*. **b** Principal component analysis (PCA) with environmental variables from 240 occurrence data points

Observed measures of niche-overlap and identity (*D* = 0.433 and *I* = 0.708, see Fig. 6a) were lower than the null distributions for var. *chinensis* vs. var. *deliciosa*, suggesting high niche differentiation between these two varieties. In addition, more detailed discrepancies can be found in the niches of three different cytotypes (Additional file 1: Figure S4). Thus, each variety or cytotype occupied a different niche with distinct environments, which was also supported by the results of PCA analysis as based on the data from elevation and 19 bioclimatic variables (Fig. 6b).

## Discussion

### High ploidy diversity in *A. chinensis* complex

To our knowledge, this study represents the most comprehensive investigation of the ploidy variation of *A. chinensis* complex by examining 128 populations and multiple individuals per population (28 on average) across their entire geographic distribution. Var. *deliciosa* predominantly comprises tetraploids and hexaploids, whereas var. *chinensis* consists of diploids and tetraploids, which is largely consistent with the results of Li et al. [25] where only the populations from Hunan and Guizhou provinces, China, were studied. For the *A. chinensis* complex, hexaploids were centered in the western portion of their ranges and diploids and tetraploids in the eastern portions. For var. *deliciosa*, the most frequent and widespread cytotype is hexaploid, followed by tetraploid. For var. *chinensis*, diploid is geographically most widespread followed by tetraploid. This is the first study to detect octoploids in var. *deliciosa*. In addition, natural triploid var. *chinensis* is described for the first time. The low frequency of triploids within diploid-tetraploid populations suggest the probability of interaction between diploids and tetraploids in the contact zone and highlights the potential for contemporary gene flow from diploid to polyploid populations.

In total, eight different mixed-ploidy populations were observed, including two majority cytotypes (2x + 4x, 4x + 6x,) and six minority cytotype (2x + 3x, 2x + 3x + 4x, 2x + 4x + 6x, 2x + 3x + 4x + 6x, 2x + 4x + 6x + 8x, 6x + 8x) (Fig. 1). The coexistence of cytotypes at the infraspecific or even intrapopulation levels has been documented for many plant species [32]. The maintenance of multiple cytotypes within the same sites may be explained by the divergence in pollinator spectra and/or visit frequency, different flowering time, and other factors associated with reproductive isolation [25]. For example, our field investigation shows diploids often flower slightly earlier than tetraploids and hexaploids, which suggests a reproductive barrier between cytotypes in the overlapping niches. The overall cytotype distribution pattern may arise from divergent adaptive abilities to the local environment. Generally, polyploid species have been suggested to be more adaptive to new ecological conditions than the ancestral diploid [14, 33, 34]. The overall geographical pattern of cytotype segregation in the species complex revealed in the present study implies kiwifruit species enhanced their adaptability to harsh climate conditions (e.g. colder climate) by reaching a higher ploidy level [25].

### Multiple origins of polyploids

Despite autopolyploids being challenging to identify, several lines of evidence, such as isozymes and DNA sequences derived from the polygalacturonase gene, suggest that tetraploid var. *chinensis* is autoploid and hexaploid var. *deliciosa* is alloploid [35, 36]. The polyploids of the *A. chinensis* complex may experience multiple origins regardless of whether they are autopolyploids or allohexaploids. In this study, 31 haplotypes were detected in *A. chinensis* complex, of which 19 were found in diploids only, six were found in polyploids (tetraploid or hexaploid) only, and six were shared by different ploidy. Moreover, none of these polyploidy haplotypes were clustered together in the phylogenetic analysis (e.g. H16 and H17 in Fig. 3a). If we assume that diploids produced the tetraploids with identical cpDNA haplotypes (e.g. H16, H10, H7, and H26 in Fig. 3a), each haplotype has only evolved once (without parallel evolution). In that case, we can conclude that the tetraploids in the *A. chinensis* complex had experienced at least four independent origins in the evolutionary history of this species.

Previous studies have proposed that another hypothesis of sharing multiple haplotypes between diploids and tetraploids resulted from the hybridization among the different ploidy race [37, 38], where plastids and nuclear genes can be transferred to tetraploids by unreduced gametes or triploid bridges [39–42]. In the present study, a few triploid individuals were found in the mixed 2x-4 × populations. This finding implies that the hybridization may exist in the *A. chinensis* complex among the different ploidy races. Our data are still insufficient for distinguishing between independent vs. hybrid origins. Futher study is needed to test this hypothesis with a more in‐depth analysis and an increased marker density. However, multiple independent origins of both autopolyploids and allopolyploids through new polyploidization events, rather than hybridization have been widely reported in previous studies [17, 43–47]. For example, multiple independent origins were observed in the *Allium przewalskianum* based on the same methods [17]. In the present study, whatever the type of origin, our data reveal the multiple origins of polyploids in the *A. chinensis* complex.

### Genetic divergence between var. *chinensis* and var.* deliciosa*

In this study, 31 haplotypes were obtained by sequencing the cpDNA of 50 populations of *A. chinensis* complex, and the genetic diversity (*H*_T_) at the species level was 0.884. Among these haplotypes, the chloroplast genetic diversity (*H*_T_) of var. *chinensis* was 0.920, which was significantly higher than that the relict plants in this region, such as *Cercidiphyllum japonicum* (0.757) [48], *Davidia involucrata* (0.882) [49], and *Eupleea pleiosperma* (0.893) [50]. However, the genetic diversity (*H*_T_) of var*. deliciosa* was 0.552, which is significantly lower than that of other species distributed in the same region. Although the evolutionary rate of mutation of the chloroplast genome is generally slow and the level of intraspecific genetic diversity low [51], there are still many genetic variants accumulated. Var. *chinensis* originated about 4.43 million years ago in China, whereas var*. deliciosa* originated about 2.22 million years (Fig. 3). Moreover, var. *chinensis* has a wide distribution with high environmental heterogeneity, which results in an increased probability of isolation, drift, and mutation [50]. Although var*. deliciosa* is also widely distributed, its suitable habitats are relatively less than those for var. *chinensis* (see below). In addition, previous studies have also found that widespread species tend to have a higher level of genetic diversity than local species [48]. The same distinction also exists in their genetic structure: var*. chinensis* has a clear genetic structure (*G*_ST_ / *N*_ST_ = 0.490/0.591, *P* < 0.01), while var*. deliciosa* does not have such genetic structure (*G*_ST_ / *N*_ST_ = 0.312 /0.252, *P* > 0.01). Although the above results indicate a significant genetic divergence between the two varieties. The finding of the lineage 1 haplotypes in the sympatric region suggested potential hybridization. In particular, the haplotype H16 was shared by the two varieties, indicating that genetic mixture occurred between these two varieties. Hybridization and introgression have been considered to be common in the evolution of *Actinidia* [52, 53].

### Pliocene intraspecific divergence and population dynamic history

Global climate cooling is hypothesized to be a key trigger for climate change in East Asia, which enhances the monsoon climate in East Asia simultaneously [54, 55]. The rapid rise in elevation on the eastern edge of the Qinghai-Tibet Plateau in the mid-Pliocene (c. 3.6 Ma) led to dramatic changes in the landscape of southwestern China [55, 56]. The estimated divergence time between the two major lineages of the *A. chinensis* complex was 4.43 Ma (95% CI: 2.81–6.39; see node A in Fig. 3), which coincides with the accelerated rate of cooling of the early Pliocene of the Tertiary period [10]. Therefore, we suggest that climate and geological changes since the Pliocene may have played important roles in habitat fragmentation and the geographical barrier to gene flow, which in turn led to early genetic differentiation of the *A. chinensis* complex. Such isolation and differentiation were also used to explain similar patterns of east–west differentiation in other plants from subtropical China such as *Primula obconicain* [57], *Cyclocarya paliurus* [58], and *Quercus acutissima* [59].

The BBM result of cpDNA and the distribution of an ancient haplotype (H12) revealed that the most ancient area of var*. chinensis* and eastern refugium is located in Fujian hilly region (Figs. 2 and 4). The moist and warmer climate conditions in Fujian hilly region enhance the survival of warm-temperate deciduous forest species since the LGM [1]. Restricted to Fujian hilly region in the LIG period, var*. chinensis* then rapidly migrated eastward, westward, and northward from this refugial area, reaching the vast regions since the LGM. In addition, seed dispersal mechanism (animal dispersal), polyploidization, and interspecific/intraspecific hybridization may also have contributed to its rapid spread and expansion [52].

The ENMs analysis based on paleoclimate shows that the distribution of the *A. chinensis* complex has experienced Last Interglacial contraction and Last Glacial Maximum expansion, which is mutually supported by the results of population dynamic analysis. This dynamic pattern is the same as that of *Pinus kwangtungensis* [60] and *Emmenopterys henryi* [9], which belong to the region of subtropical China. The *A. chinensis* complex is a montane deciduous broad-leaved plant distributed throughout the subtropical zone of China with an elevation of 200–2500 m [26]. Previous studies have that changes in temperature can cause montane species to migrate up and down mountainous slopes [61, 62]. In the LIG period, the temperature was 2 °C higher than that of the present [63], in which case montane species will migrate to higher elevation, and the distribution of species will be more fragmented or even disappear because of theabsence of appropriate habitat. However, in the LGM period of lower temperature, these species will migrate to lower elevations in more suitable habitats with population expansion, and groups on different mountain may even reunite. Moreover, polyploidization would promote a broader niche, which has been verified in previous studies [64–67]. In this study, the ecological niche differentiation between var*. chinensis* and var*. deliciosa* has been confirmed. Our field survey data directly show that the elevational distribution range of var. *deliciosa* (including tetraploid of var*. chinensis*) is higher than that of var*. chinensis* (mainly diploid). Therefore, based on the MDA analysis of demographics, two groups (group E4x and M4 × 6x, Additional file 1: Figure S3) including polyploid individuals show a clear history of population expansion.

Polyploids often differ in physiological and life-history characteristics that may confer adaptive advantages compared with their diploid ancestors. Previous studies have also revealed a causal relationship between polyploidy and population expansion or ecological radiation in plants [68–70]. For example, Ramsey [71] compared the adaptability to the environment for *Achillea borealis* with different ploidy and found that the adaptability to the environment of hexaploid *A. borealis* was stronger than that of tetraploid and more than 70% of the fitness advantage was produced in the polyploidization process. In the present study, the polyploid populations (group E4x and group M4 × 6x) showed a historical population expansion (Additional file 1: Figure S3), but the population expansion was not observed in diploid populations (group E2x and M2x). The results of niche overlap and identity tests between three main ploidy (2x, 4x, and 6x) indicate that the environmental niche of hexaploid var. *deliciosa* was distinguishable from both diploid and tetraploid *A. chinensis* (Additional file 1: Figure S4). In particular, the hexaploid var. *deliciosa* colonized westward to the areas with cooler and slightly drier environments compared with their diploid parents (Fig. 2). Although the ecological niche of tetraploid overlaps with that of diploid, the tetraploid usually occupied higher elevations in the sympatric region. This ecological radiation pattern is revealed in other plants such as *Aster amellus* [72], *Larrea tridentata* [73], or *Senecio carniolicus* [74] where higher ploidy cytotypes could experience niche expansion whereas low levels were unable to occupy their full potential niche [32]. Thus, polyploidization appears to have played an important role in the historical demography of the *A. chinensis* complex through adaptation to environmental changes.

## Conclusions

We sampled populations of the *A. chinensis* complex across its geographic distribution in subtropical China to study its cytotype distribution and phylogeography. The most frequent and widespread cytotypes were diploids followed by tetraploids and hexaploids, whereas triploids and octoploids were only observed in a few populations. The ENMs and BBM results suggested that Pliocene and Plio-Pleistocene climatic fluctuations and vicariance played key roles in shaping the current population structure and historical demography of the *A. chinensis* complex. This is consistent with the hypothesis that tracking suitable habitat by expansion or contraction would be the main pathway responding to climate changes for the species with a conservative ecological niche. Moreover, the polyploidization of the *A. chinensis* complex also appears to have played an important role in the historical demography of adaptation to environmental changes.

## Methods

### Plant sampling

From 2015–2018, 3586 samples were collected from 128 populations representing the species complex *A. chinensis* Planch. including the vars. *chinensis* and *deliciosa*. Another variety, *A. chinensis* var. *setosa*, narrowly distributed in the Taiwan mountains was not included in the present study because its taxonomic status is controversial. The taxonomy of the varieties is adopted from Li et al. [20, 75], which is based mainly on morphological characteristics such as the hair types covering flowering branchlets and fruits (var. *chinensis*: finely tomentose; var. *deliciosa*: brown strigose hairs), the presence of over-wintering buds (var. *chinensis*: exposed; var. *deliciosa*: buried in the bark) and the color of the flesh of the mature fruit (var. *chinensis*: mostly yellow or yellow-green; var. *deliciosa*: mostly green). Field collection followed the ethics and legality of the local government and was permitted by the government. The formal identification of the plant material was undertaken by the National *Actinidia* Germplasm Repository (NAGR) of China, and voucher specimens were deposited at NAGR. Each population consisted of either var. *chinensis* or var. *deliciosa*, and there were some populations containing both varieties as based on morphology in the sympatric regions. For each population, 6 to 72 individuals were sampled (Additional file 2: Table S1). To avoid collecting closely related individuals, sampled individuals were separated by ca. 50–100 m. For the subsequent cpDNA analysis, the fresh leaves were dried in silica gel in the field and then stored at -80 °C. One-year-old shoots were also collected in the field, providing fresh leaves for the ploidy level identification in the laboratory. These shoots were stored at 4 °C in a refrigerator for at least 30 days to overcome dormancy and then placed in fresh water at room temperature to stimulate bud breakage for the collection of fresh leaf tissue. The latitude, longitude, and elevation of each sampled individual were recorded with a global positioning system.

### Ploidy analysis

The ploidy level of individuals were determined by estimating its relative DNA content with FCM. The detailed FCM method in this study was performed as in Li et al. [25]. Briefly, fresh leaves of the sample were chopped with leaves of the internal standard in 0.5 mL of ice-cold Otto I buffer (solution A of the High-Resolution Kit, Partec, Germany) and then were filtered through a 30 µm nylon sieve, followed by the addition of 1 mL Otto II buffer (4 µg/mL 4′,6-diamidino-2-phenylindole [DAPI]; solution B of the kit) held at 4 °C. Five minutes later, these samples were used to estimate the ploidy levels on a CyFlow Ploidy Analyser (Partec, Germany) automatically. Relative fluorescence intensity of a diploid var*. chinensis* cultivar, ‘Hongyang’ (2n = 58), whose chromosome number had been determined previously from chromosome counts and the estimated genome size of 750 Mb [76], was used as an internal standard for each measurement [25].

### DNA isolation, amplification, and sequencing

For chloroplast DNA analysis, 50 out of 128 populations were selected to cover the entire geographic distribution of the *A. chinensis* complex. A modified CTAB method [77] was used to extract total genomic DNA from 608 individuals (Additional file 2: Table S2) which were selected in 50 population as evenly as possible by the predominant ploidy level (rare ploidy levels were excluded), then 1% agarose gels and NanoDrop 8000 (Thermo Fisher Scientific, Waltham, MA, USA) were employed to examine the quality and concentration of genomic DNA, respectively.

To identify chloroplast DNA (cpDNA) regions with sufficient variation, 12 individuals of var*. chinensis* and 12 var*. deliciosa* were randomly selected to undertake the preliminary screening of primer pairs for eight intron regions: *ndh*F*-rpl*132*, rps*16*-trn*Q*, trn*E*-trn*T*, psb*A*-trn*H*, rpl*16*, trn*L*-trn*F*, trn*L*-trn*T*,* and *trn*D*-trn*T (Additional file 2: Table S6). Finally, three consistently amplified and variable non-coding intergenic spacer (IGS) regions *ndh*F*-rpl*132*, rps*16*-trn*Q, and *trn*E*-trn*T [21], were selected for further analysis. A total of 608 individuals from all 50 populations of the *A. chinensis* complex were sequenced. Amplification was carried out in a volume of 20 μL reaction solution containing 10 μL 2 × Taq PCR MasterMix ( Bioteke), 0.5 μL each primer (0.2 μM), 1 μL template DNA (ca. 50–100 ng) and 8 μL ddH2O. The thermo-cycling conditions for PCR are given as follows: 94 °C, 4 min; 35 × (94 °C, 30 s; 56 °C, 60 s; 72 °C, 60 s); and 72 °C, 10 min. PCR products were sequenced in an ABI 377XL DNA sequencer (Applied Biosystems). All haplotype sequences identified in this study were submitted to GenBank (MT812986-MT813020). For subsequent analysis, *A. eriantha* and *A. polygama* were used as the outgroup and these chloroplast sequences were downloaded from GenBank (KY100979.1 and KX345297.1).

### Analyses of genetic diversity and population structure

Three cpDNA fragments were aligned separately and trimmed in MEGA 6 [78] and combined into a single dataset with the online tool FaBox [79] for the phylogeographic analyses. During the analyses, indels (gaps) were treated as a single mutation event and coded as substitutions A or T (a third G or C were used when three kinds of mutations coexisted). The number of cpDNA haplotypes, haplotype diversities (*H*_d_), and nucleotide diversities (π) for each variety were calculated with DnaSP 5.10 [80]. Haplotype distribution maps were constructed with ArcGIS 10.3. Total genetic diversity (*H*_T_) and within-population diversity (*H*_S_) were calculated with PermutCpSSR 2.0 [81].

The phylogeographic structure was inferred by comparing population differentiation for phylogenetically ordered (*N*_ST_) and unordered (*G*_ST_) haplotype with PermutCpSSR 2.0. A test of significance comprising 1000 permutations was used to determine if *N*_ST_ > *G*_ST_. A significantly higher *N*_ST_ than *G*_ST_ usually indicates the presence of phylogeographical structure. The geographically and genetically distinguishable groups and the potential barriers between groups were analyzed with SAMOVA 2.0 [82]. Various SAMOVA were run, increasing the number of *K* groups until the percentage of explained variance among groups reached a limit (*K* = 2–20). In addition, a molecular variance (AMOVA) analysis was performed using Arlequin v3.5 [83] with significance tested under 1023 permutations to test genetic differentiation among populations and between varieties. Finally, estimates of pairwise *F*_ST_/(1 − *F*_ST_) were regressed against the pairwise natural logarithm of the geographic distance by using a Mantel test with 999 random permutations in GENALEX 6.5 to assess IBD patterns among the populations [84, 85]. Measures of pairwise *F*_ST_ distances were calculated with Arlequin, and the pairwise natural logarithms of geographic distance were calculated with GENALEX 6.5.

### Haplotype relationships and divergence time estimation

To determine phylogenetic relationships among haplotypes, median-joining networks were constructed with NETWORK v5.0 [86]. *Actinidia eriantha* and *A. polygama* as the outgroup were included in haplotype network construction as well as molecular dating analysis. We used Bayesian phylogenetic inference on all haplotypes (sequence without recoding) with Markov chain Monte Carlo (MCMC) methods. A strict clock model in BEAST2 v2.5.0 was performed to estimate divergence time among lineages [87]. A GRT model was selected from the result of Akaike Information Criterion (AIC) with jModelTest v 2.1.10 test [88]. As a tree prior, the Yule model was specified. Two calibration points from previous studies of *Actinidia* [53] were used to constrain the nodes with a normal distribution prior. One point is the estimated split between *A. polygama* and the other two species (19 Myr, 95%CI: 14–24), and another is the estimated split between *A. eriantha* and *A. chinensis* (11 Myr, 95%CI: 4.7–17.3). The Monte Carlo Markov chain runs were performed every 1 × 10^9^ generations, with sampling every 1 × 10^5^ generations, following a burn-in of the initial 10% cycles. The convergence and effective sample size (ESS) > 200 for all parameters were assessed with Tracer version 1.7.1. After discarding the first 25% trees as burn-in, the rest of the trees were summarized in a maximum clade credibility tree with treeAnnotator version 2.6.0. Finally, the maximum clade credibility tree was visualized in FigTree version 1.4.3 (available at http://tree.bio.ed.ac.uk/software/figtree/).

### Demographic history analyses and ancestral geographical area reconstruction

The mismatch distribution analysis was conducted with Arlequin 3.5 [83] to test whether each group has undergone recent demographic or spatial population expansion events. Multimodal mismatch distributions of pairwise differences between individuals are expected for populations at demographic equilibrium with a relatively stable size over time, whereas unimodal distributions are expected for the population that has experienced recent demographic expansions [89]. The goodness-of-fit of observed mismatch distributions to the theoretical distributions under a model of sudden expansion was tested with the raggedness index [90]. The significance of the raggedness index was obtained by examining the null distribution of 1000 coalescent simulations of these statistics. Small values of the raggedness index suggest sudden expansion, whereas high values of the raggedness index suggest stationary or genetic bottleneck. Second, for expanding lineage, the expansion parameter (τ) and its 95% confidence interval were converted into generation time (T) since expansion following the equation: T = τ/2μ [91], where μ is the neutral mutation rate of the entire cpDNA sequences per generation. The value for μ was calculated as μ = uk, where u is substitution rate in substitutions/site/generation (s/s/g) (here, 1.0 × 10^–8^; refer to Gaut [92]), k is the average sequence length of the cpDNA region under this study (here, 1,854 bp), the expansion time was calculated by assuming a generation time of 7 years for *A. chinensis* under natural conditions [53]. Finally, we also calculated Tajima’s *D* [93] and *F*_S_ statistics of *F*_u_ [94] using Arlequin 3.5 [83], which is based on 1000 random permutations. The mismatch distribution of the observed number of nucleotide differences between pairs of DNA sequences was computed with DnaSP 5.10 [80].

Ancestral range reconstruction was conducted to estimate possible historical patterns of geographical distribution for *A. chinensis* complex using the Bayesian binary MCMC method (BBM) implemented in RASP v.4.0 [95]. For BBM analysis, 10,001 BEAST-generated trees and consensus tree without the outgroup were used as topology input, excluding the first 1000 as burning. In the ancestral area reconstruction, the geographical areas of the *A*. *chinensis* complex were defined four biogeographical regions: EA, East; SO, South; CE, Central; and WE, West (Fig. 4a) [52]. Ten BBM chains were run for 100,000 generations with a sampling frequency of 100. F81 was used as a state frequencies model in accordance to the AIC.

### Ecological niche models

To study the niche differentiation and distributional changes for each variety, the Maxent Version 3.4.0 program [96, 97] was used to undertake to test of the ecological niche models and projected their potential distributions during three periods: the present, the Last Glacial Maximum (LGM, 21kya), and the Last Interglacial (LIG,120–140 kya). The geographic distribution data were obtained from our field collection and the Chinese Virtual Herbarium (CVH, available at http://www.cvh.ac.cn/). For all CVH records, the taxonomic identity was verified by specimen images and records of specimens taken from cultivated plants removed to ensure that geolocations were consistent with the known distribution. After removing duplicates, 150 sites for var*. chinensis* and 90 sites for var*. deliciosa* were identified. The current and past environmental datasets of 19 bioclimate variables with spatial resolutions of 2.5 arc minute were downloaded from the WorldClim database [98]. To avoid over-fitting [99], strongly correlated bioclimatic parameters, according to Pearson’s coefficient (|r|> 0.8) with the Perl program ENMTools v.1.3 [100], were excluded. Finally, six bioclimatic variables (Additional file 2: Table S7) were retained for ecological niche model construction with Maxent. The model quality was assessed by cross-validation comprising 100 replicates with 25% of the data for model testing, and the maximun number of background points was 10,000. The accuracy of each cross-validation test was evaluated with the area under the ROC curve (AUC) [101].

### Ecology divergence analysis

To measure niche divergence, the environmental niches of the two varieties were compared using niche overlap and identity tests, in which the difference between the actual niches was contrasted with null models generated from randomly reshuffled occurrence points [102]. Niche identity was calculated with Schoener’s *D* similarity index [103] and Warren’s *I* [102] implemented in ENMTools version 1.3 [100, 102]. Both Schoener’s *D* and Warren’s *I* ranged from 0 (no niche overlap) to 1 (identical niches). One hundred pseudoreplicates of shuffling were conducted to generate null models for these statistics, and tested for significance; histograms were drawn using R 3.6.0 (http://www.rproject.org/). In addition, the same tests and analyses were also carried out for the three main different level ploidy (2x, 4x, and 6x) populations.

To better understand and visualize the similarities and differences between the environments in which var. *chinensis* and var. *deliciosa* occur, principal components analyses (PCA) were conducted with the elevation variable and 19 other bioclimatic variables from WorldClim [98, 104]. The same 240 occurrence data points used for ENMs were extracted values in ArcGIS 10.3. Before the PCAs analysis, the matrices of environmental values were standardized simultaneously. The distributions of species in environmental space were visualized in R 3.6.0.

## Supplementary Information

**Additional file 1: Figure S1.** Schematic diagram of flow cytometric histogram of DAPI-stained nuclei of six type of ploidy levels (2x, 3x, 4x, 6x, and 8x) of the *A. chinensis* complex analyzed simultaneously with the internal standard *Actinidia chinensis* cv. ‘Hongyang’. **Figure S2.** Results of spatial analysis of molecular variance analysis (SAMOVA, *K* = 2-20) on the *A. chinensis* complex populations in subtropical China. *K* refers to the number of predefined groups used in the analyses. **Figure S3.** Observed and expected mismatch distributions for different groups of the *A. chinensis* complex. (a) group E2x; (b) group E4x; (c) group M2x; (d) group M4x6x. The solid line indicates observed distribution; The dashed line represents theoretically expected distribution under a population expansion model. **Figure S4.** The results of niche-identity tests by comparing the niches of three main ploidy level populations. (a) 2x vs. 4x (b) 2x vs. 6x (c) 4x vs. 6x.

**Additional file 2: Table S1.** Characteristics of sampled populations and ploidy levels of the *A. chinensis* complex. **Table S2.** List of ploidy level and haplotype for 608 individuals in cpDNA analysis. **Table S3.** Description of haplotypes in the *A. chinensis *complex from three combined chloroplast DNA fragments. **Table S4.** Genetic characteristics of cpDNA for 31 var. *chinensis* populations and 25 var. *deliciosa* populations. **Table S5.** Results of AMOVA testing of the *A. chinensis* complex. **Table S6.** List of primers for the eight cpDNA loci. Table S7 The six environmental variables used for ecological niche modeling.

## Data Availability

All sequences of *A. chinensis* complex generated during the current study are available in the NCBI GenBank database under accession numbers MT812986-MT813020.

## Abbreviations

ENMs: Ecological niche models
LIG: Last Inter Glacial
LGM: Last Glacial Maximum
FCM: Flow cytometric measurement
MDA: Mismatch distribution analysis
SSD: Sum of square deviations
HRI: Harpending’s raggedness index
IBD: Isolation-by-distance
RASP: Reconstruct Ancestral State in Phylogenies
BBM: Bayesian Binary MCMC
AUC: Area Under the Curve
LDD: Long-distance dispersal
NAGR: The National *Actinidia* Germplasm Repository
DAPI: 4′,6-Diamidino-2-phenylindole
CVH: Chinese Virtual Herbarium
MCMC: Markov chain Monte Carlo
AIC: Akaike Information Criterion
PCA: Principal components analyses
