# Supplementary material for: Cytotype distribution and chloroplast phylogeography of the Actinidia chinensis complex
Source: BMC Plant Biol. 2021 Jul 6;21:325. doi: 10.1186/s12870-021-03099-y (PMC8259359; doi:10.1186/s12870-021-03099-y)
Supplement: Supplementary file 1 — Additional file 1: Figure S1. Schematic diagram of flow cytometric histogram of DAPI-stained nuclei of six type of ploidy levels (2x, 3x, 4x, 6x, and 8x) of the A. chinensis complex analyzed simultaneously with the internal standard Actinidia chinensis cv. ‘Hongyang’. Figure S2. Results of spatial analysis of molecular variance analysis (SAMOVA, K = 2-20) on the A. chinensis complex populations in subtropical China. K refers to the number of predefined groups used in the analyses. Figure S3. Observed and expected mismatch distributions for different groups of the A. chinensis complex. (a) group E2x; (b) group E4x; (c) group M2x; (d) group M4x6x. The solid line indicates observed distribution; The dashed line represents theoretically expected distribution under a population expansion model. Figure S4. The results of niche-identity tests by comparing the niches of three main ploidy level populations. (a) 2x vs. 4x (b) 2x vs. 6x (c) 4x vs. 6x. [file 12870_2021_3099_MOESM1_ESM.pdf]

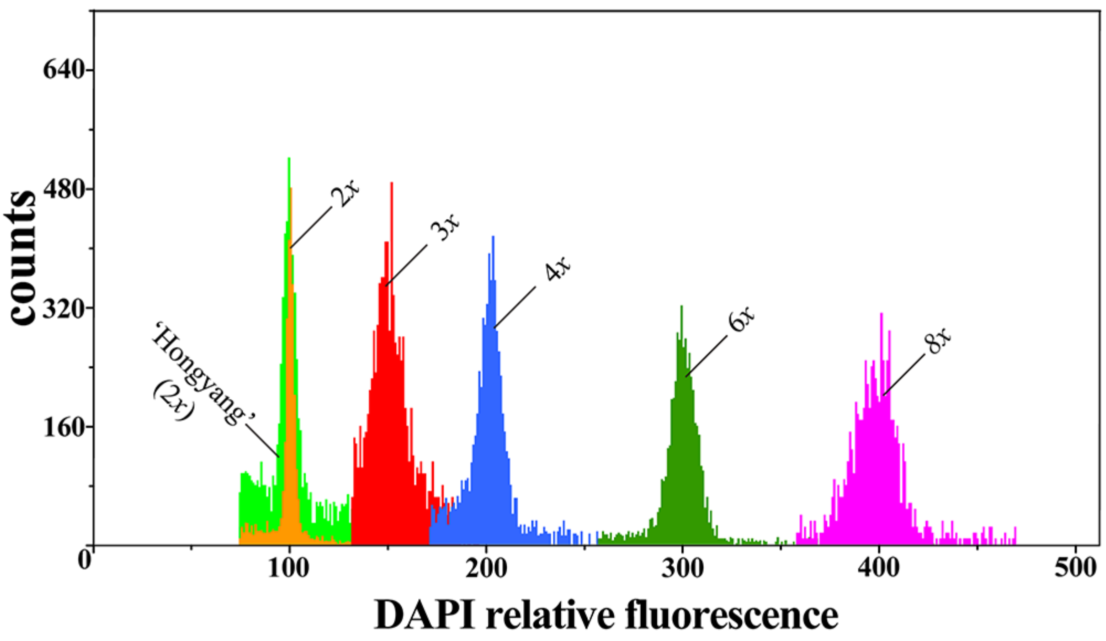


**Figure S1** Schematic diagram of flow cytometric histogram of DAPI-stained nuclei of six type of ploidy levels (2x, 3x, 4x, 6x, and 8x) of the *A. chinensis* complex analyzed simultaneously with the internal standard *Actinidia chinensis* cv. 'Hongyang'.

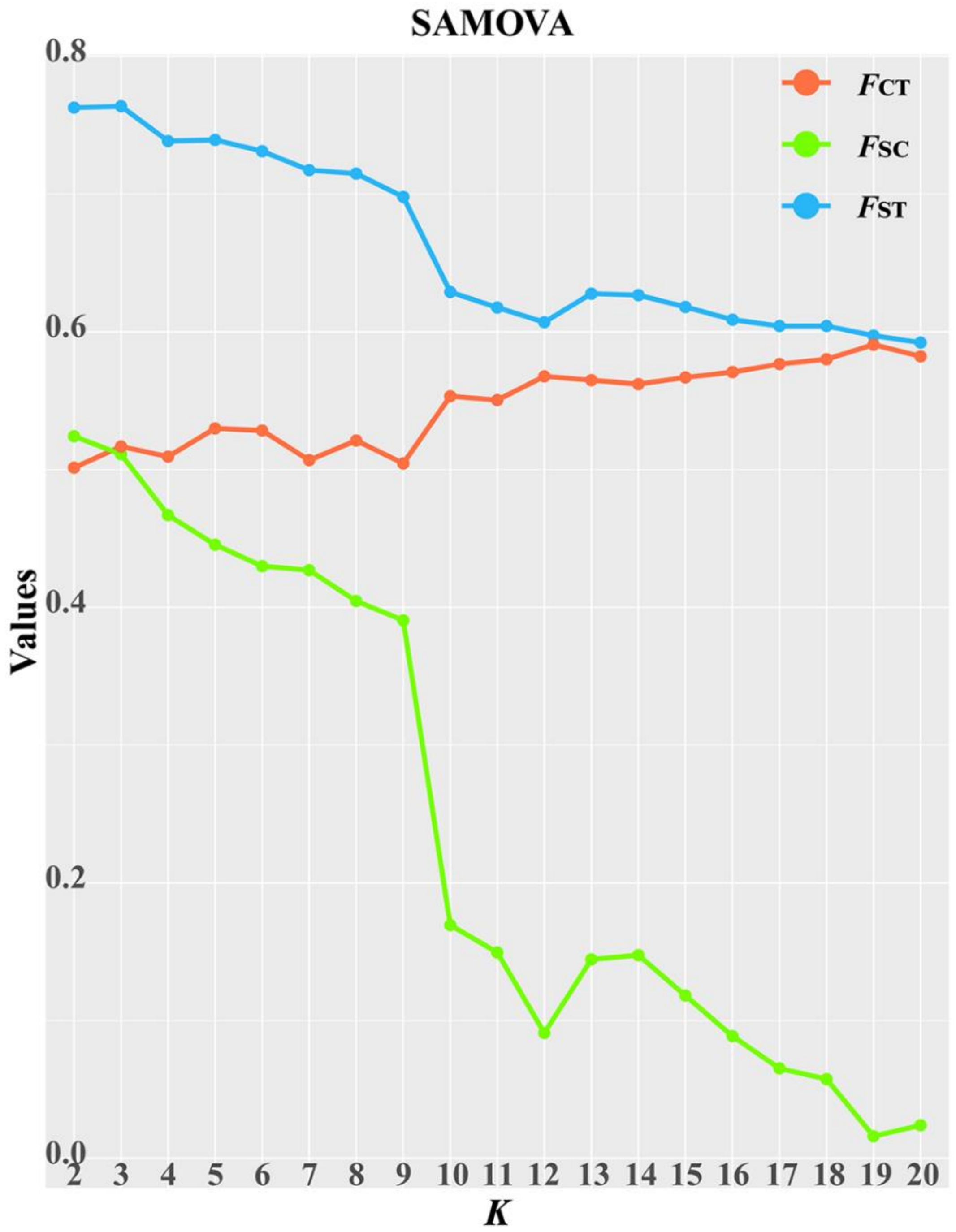


**Figure S2** Results of spatial analysis of molecular variance analysis (SAMOVA, *K* = 2-20) on the *A. chinensis* complex populations in subtropical China. *K* refers to the number of predefined groups used in the analyses.

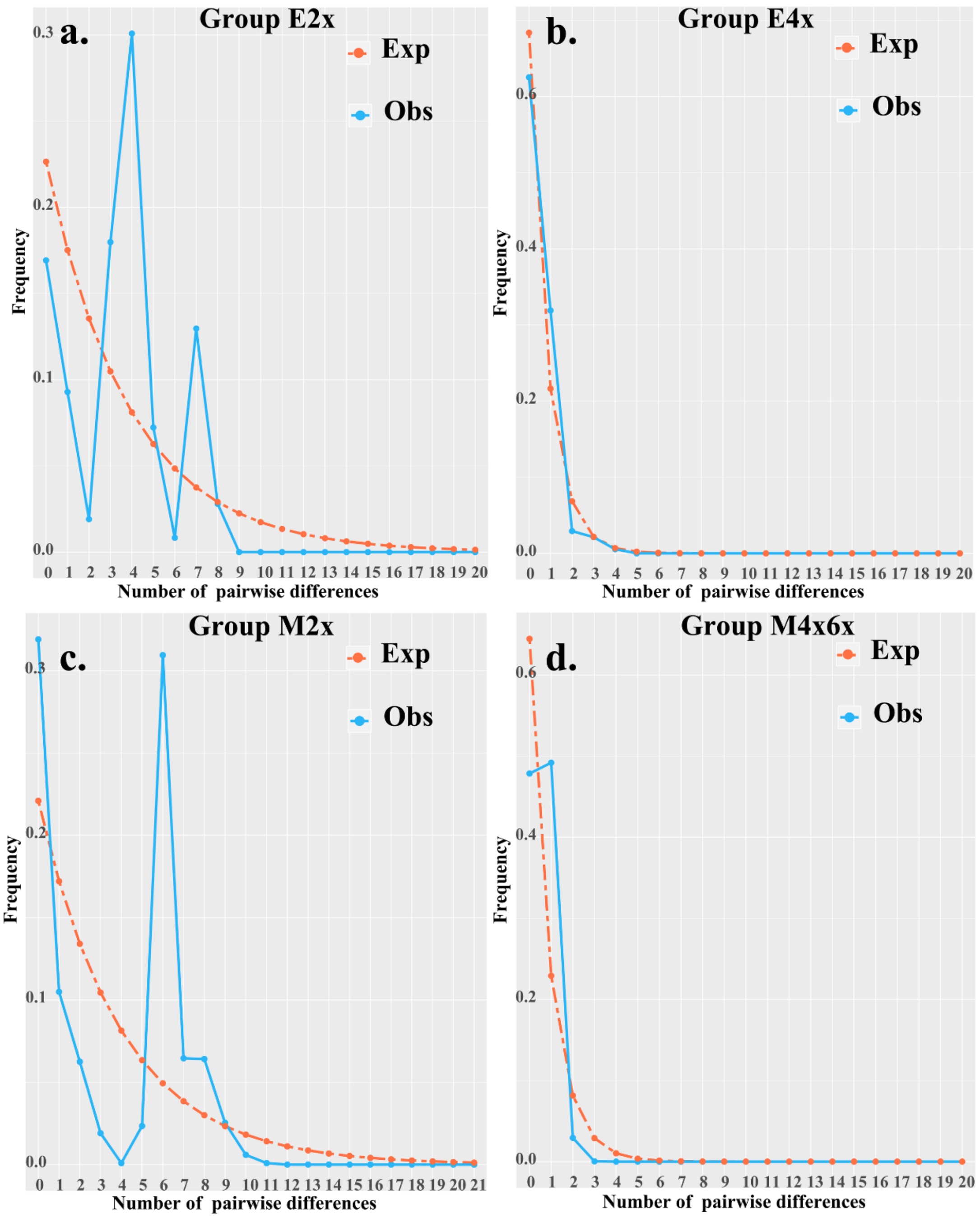


**Figure S3** Observed and expected mismatch distributions for different groups of the *A. chinensis* complex. (a) group E2x; (b) group E4x; (c) group M2x; (d) group M4x6x. The solid line indicates observed distribution; The dashed line represents theoretically expected distribution under a population expansion model.

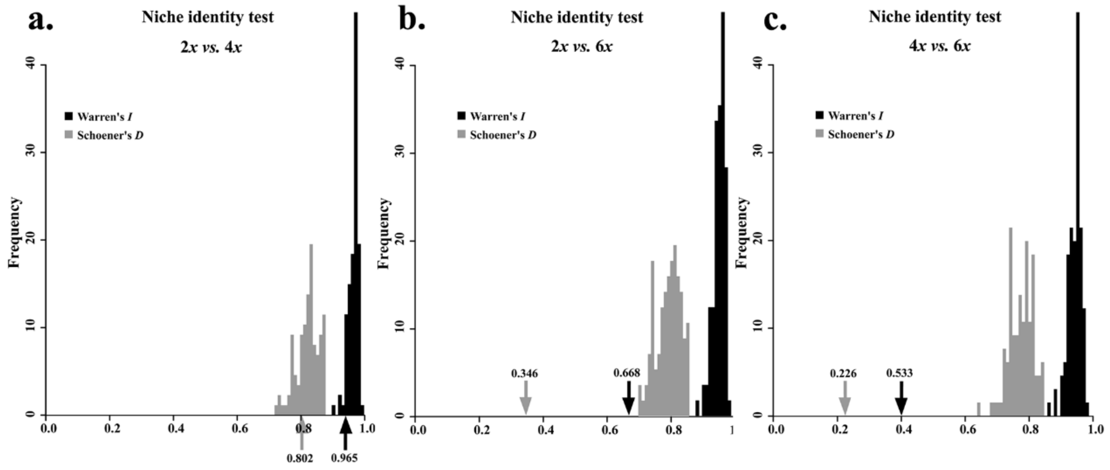


**Figure S4** The results of niche-identity tests by comparing the niches of three main ploidy level populations. (a) 2x vs. 4x (b) 2x vs. 6x (c) 4x vs. 6x
